# Correlation between muscular edema on magnetic resonance imaging versus major histocompatibility complex type Ii overexpression on muscle biopsy at diagnosis on juvenile dermatomyositis patients

**DOI:** 10.1186/1546-0096-12-S1-P90

**Published:** 2014-09-17

**Authors:** Estíbaliz Iglesias, Emilio Inarejos Clemente, Cristina Jou, Vicenç Torrente-Segarra, Lucía Riaza, Rosa Bou, Teresa Ribalta, Jordi Antón

**Affiliations:** 1 Pediatric Rheumatology, Hospital Sant Joan De Déu, Esplugues De Llobregat, Barcelona, Spain; 2 Diagnostic Imaging, Hospital Sant Joan De Déu, Esplugues De Llobregat, Barcelona, Spain; 3 Pathology, Hospital Sant Joan De Déu, Esplugues De Llobregat, Barcelona, Spain

## Introduction

Juvenile Dermatomyositis (JDM) is the most common idiopathic inflammatory myopathy in childhood. Magnetic resonance imaging (MRI) is a non invasive tool to assess muscular edema. Its ability to distinguish between active JDM patients and inactive and healthy children is well described in the literature. However muscle biopsy still remains the gold standard in JDM diagnosis. Major Histocompatibility Complex (MHC) type I is overexpressed on sarcolemma of inflammatory myopathies and it could be detectable before inflammatory infiltrate appears on conventional tecniques and remains there in spite of treatment.

## Objectives

To assess clinical characteristics, muscular MRI pattern and MHC type I overexpression on sarcolemma and sarcoplasma on muscle biopsy at diagnosis of our JDM patients among 2000 and 2013.

## Methods

We made a retrospective chart, including MRI and muscle biopsies. Muscular edema, fascia involvement and soft tissue edema were assessed by a Pediatric Radiologist on paravertebral and scapular and pelvic girdle muscles and scored as present or not and defined as patchy or diffuse. Muscle biopsies were evaluated with conventional tecniques (hematoxiline-eosine and tricromique) and MHC type I inmunohistochemical study. We evaluated perifascicular atrophy, regenerated and necrotic fibers and inflamatory infiltrated, being pathological the presence of one of the aforementioned. We study the MHC type I overexpression on sarcolemma and sarcoplasma scored as mild=1, moderate=2 and severe=3, describing as well the percentage of affected muscle fibers. The results were assessed by a neuropathologist.

## Results

23 patients were included. Demographic and clinical characteristics are summarized on table 1. MRI: 16 patients had MRI at diagnosis being pathological in 15. Thigh muscles were the most frequently affected (93% vs 86% and 79% of arm and paravertebral muscles respectively) and diffuse pattern more common than patchy one. 5 patients had soft tissue edema, 4 of these with fascia edema too. Muscle biopsy: 18 patients had muscle biopsy at diagnosis. All biopsied muscles except one were pathological on simultaneously MRI. Histological study show muscle affectation in 14 out of 18 patients but all patients overexpressed MHC type I on sarcolemma and sarcoplasma with 100% of fibers affected and a range of 50-100% and 15-100% on sarcolemma and sarcoplasma respectively. Median intensity scored on sarcolemma and sarcoplasma was 3 [1-3] and 1,5 [1-2].

## Conclusion

MRI is an important non invasive tool to evaluate muscular edema in JDM patients but MHC type I overexpression on muscle biopsy seems to be the most sensitive technique for diagnosis at this moment. All our patients overexpressed MHC type I on sarcolemma and sarcoplasma, despite some of them had normal histology.

## Disclosure of interest

None declared.

